# Case report: Recurrence of hypertension after renal artery angioplasty due to the progression of focal renal fibromuscular dysplasia

**DOI:** 10.3389/fcvm.2022.1008308

**Published:** 2022-11-17

**Authors:** Mona Hong, Yuanyuan Kang, Jianzhong Xu, Jiguang Wang

**Affiliations:** Department of Hypertension, Ruijin Hospital, Shanghai Institute of Hypertension, Shanghai Jiao Tong University School of Medicine, Shanghai, China

**Keywords:** hypertension, fibromuscular dysplasia (FMD), renal artery, progression, in-stent restenosis

## Abstract

Whether fibromuscular dysplasia (FMD) is a progressive disease, remains unclear. We reported a case of focal renal artery FMD that slowly progressed to a branching artery over a few years after the angioplasty without in-stent restenosis, which reconfirms that focal FMD is progressive and that such progression may be segmental. Stenting may be an option for young, risk factor-free patients with focal FMD.

## Introduction

Fibromuscular dysplasia (FMD) is a non-inflammatory, non-atherosclerotic vascular disease that may involve medium-sized arteries throughout the body and most commonly affects the renal arteries. When the renal artery is involved, the most frequent finding is hypertension (1). In young patients with recent onset of hypertension, percutaneous balloon angioplasty with bailout stenting is recommended as first-line therapy with the goal of curing hypertension (2). While restenosis occurs in more than 25% of patients with FMD within 1 year after balloon angioplasty (3), studies showed that the rate of restenosis after stenting in patients with atherosclerotic renal artery stenosis was about 10% and renal arteries that received balloon angioplasty developed restenosis earlier than arteries that received a stent (4).

Whether FMD is a progressive disease and the factors associated with disease progression remain unclear (5). Although several older studies suggested that FMD in the majority of patients progressed with time (6), the current expert consensus is that multifocal FMD of the carotid arteries is not a progressive disease (3), whereas focal FMD progresses remains unclear.

## Case description

**Figure 1:**
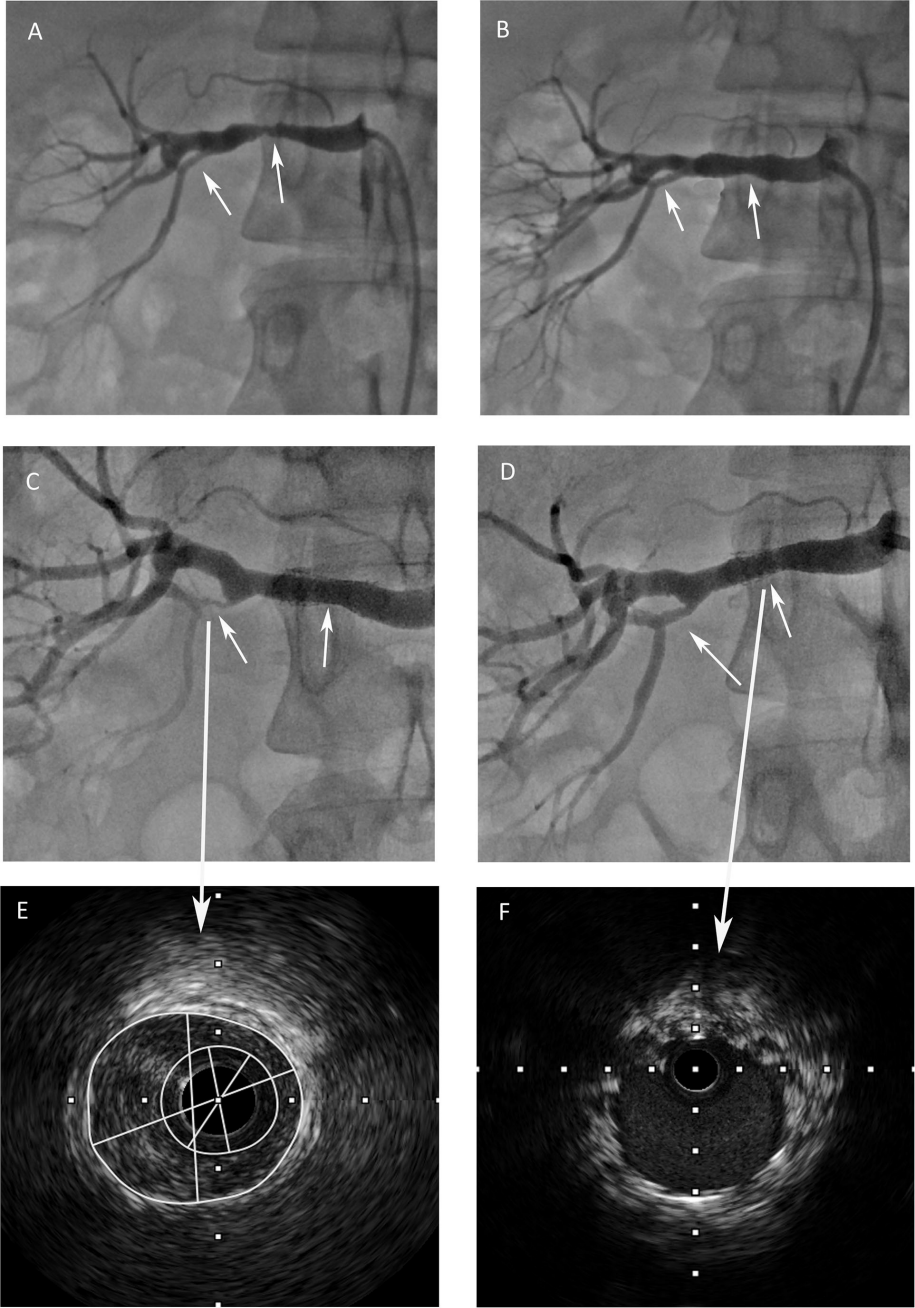
**(A)** Renal angiography revealed focal stenosis in the mid-portion of the right renal artery and the right inferior renal branch artery was normal. **(B)** After a stent was implanted into the renal artery, the right inferior renal branch artery was still normal. **(C)** Severe stenosis was found in the right inferior renal branch artery, no obvious in-stent restenosis was observed. **(D)** The lesion vessel was dilated successfully and the renal blood flow was restored after the procedure. **(E)** Intravascular ultrasound image revealed eccentric intimal thickening of the branch artery. **(F)** Intravascular ultrasound image revealed that no obvious neointimal hyperplasia in the stent.

A 31-year-old woman was referred to our hospital due to recurring elevated blood pressure for half a year. The patient first came to our hospital 8 years ago for new-onset hypertension. Investigation of probable secondary hypertension was initiated with renal disease. Renal function was preserved, with no abnormalities, and urea and creatinine levels were also normal. Kidney ultrasound showed asymmetric kidneys (right 93^*^35 mm, left 116^*^53 mm) and renal artery computed tomography angiography revealed stenosis in the mid-portion of the right kidney artery. After Takayasu and other arteritis were excluded, renal artery FMD was diagnosed. Subsequently, catheter-based renal angiography confirmed focal stenosis in the mid-portion of the right renal artery (Figure 1A, Supplementary Video 1), and a bare metal stent was implanted because of dissection after balloon dilation (Figure 1B, Supplementary Video 2). The blood pressure returned to normal (120–130/60–70 mm Hg) without any antihypertensive drugs after the procedure. The patient was recommended dual antiplatelet therapy for half a year. During the past 8 years, she monitored her blood pressure regularly, and it remained normal. She noticed 6 months ago that her blood pressure gradually increased, peaking at 180/120 mm Hg. She came to our hospital again. Biochemical tests revealed significantly elevated plasma renin activity (5.9 ng/ml/hour) and aldosterone levels (1,005.35 pg/ml) with hypokalemia (2.7 mmol/L). Then in-stent restenosis was suspected, so she was admitted to the ward for further examination. Renal angiography was performed again. However, no obvious in-stent restenosis was observed, but severe stenosis was found in the right inferior renal branch artery (Figure 1C, Supplementary Video 3), which was totally normal 8 years ago (Figures 1A,B). Intravascular ultrasound (IVUS) images revealed that eccentric intimal thickening caused stenosis of the branch artery (Figure 1E, Supplementary Video 5) and no neointimal hyperplasia in the stent (Figure 1F, Supplementary Video 6). Balloon angioplasty was performed for the branch artery and the lesion vessel was dilated successfully (Figure 1D, Supplementary Video 4). One week after the procedure, the patient was normotensive without any antihypertensives. At 1-year follow-up, the patient's blood pressure remained normal.

## Discussion

We reported a case of focal renal artery FMD that slowly progressed to a branching artery over a few years after the angioplasty without in-stent restenosis, which reconfirmed that focal FMD was progressive and that such progression may be segmental. Stenting may be an option for young, risk factor-free patients with focal FMD.

In the presented case, the renal arterial flow in the right kidney was adequate, only the branch artery blood flow was reduced. The diagnosis of renovascular hypertension was supported by renin angiotensin aldosterone system activation. The plasma renin activity and aldosterone levels were increased with hypokalemia. One similar case has been reported previously (7).

The expert consensuses recommend that balloon angioplasty without stenting is currently the first-line revascularization technique in FMD-related renal artery stenosis (2, 3). While there is no evidence that renal artery balloon angioplasty alone is superior to the stent in patients with FMD. The mechanisms of restenosis in stented lesions differ from those in balloon-dilated lesions. In balloon-dilated lesions, late constriction of the external elastic membrane after angioplasty plays a more significant role in causing restenosis than dose neointimal proliferation which is the main cause of restenosis in the stent. Since most patients with FMD undergo balloon dilation alone, the restenosis rate after stenting is unknown. In our focal FMD case, no significant neointimal hyperplasia occurred after stenting. The possible reasons were that the patient was young, had no smoking history, no diabetes, no dyslipidemia, and blood pressure returned to normal after stenting. The absence of the above risk factors may be the possible reason for the absence of neointimal hyperplasia in the stent.

Whether FMD is a progressive disease and the factors associated with disease progression remain unclear (5). It is the consensus of US experts that progression in multifocal FMD is an uncommon occurrence (3). Whether focal FMD will progress is uncertain, the progress of focal FMD to multifocal FMD has been reported recently (8). The present case is the first to report focal renal artery FMD that slowly progressed to a branching artery over a few years, with no progression at the original lesion. This observation reconfirms that focal FMD is progressive and that such progression may be segmental. In addition, IVUS images, in this case, revealed intimal hyperplasia leading to the progression of the renal branch artery. Takayasu arteritis was further ruled out because Takayasu arteritis was more often characterized by adventitial hyperplasia (9), and Takayasu arteritis was more prone to in-stent restenosis than to progression in other artery segments.

In conclusion, focal FMD is a progressive disease with a segment of progression. Stenting may be an option for young, risk-free patients with focal FMD.

## Data availability statement

The original contributions presented in the study are included in the article/Supplementary material, further inquiries can be directed to the corresponding author.

## Ethics statement

Ethical review or approval was not required for the study on human participants in accordance with the local legislation and institutional requirements. The patient provided their written informed consent to participate in this study. Written informed consent was obtained from the individuals for the publication of any potentially identifiable images or data included in this article.

## Author contributions

JX performed the intervention together with YK. MH drafted the manuscript. JX and JW contributed to manuscript design, drafting, and critical revision. All authors have critically read and reviewed this article, approved the version to be published, and agreed to be accountable for all aspects of the work in ensuring that questions related to the accuracy or integrity of any part of the work are appropriately investigated and resolved.

## Conflict of interest

The authors declare that the research was conducted in the absence of any commercial or financial relationships that could be construed as a potential conflict of interest.

## Publisher's note

All claims expressed in this article are solely those of the authors and do not necessarily represent those of their affiliated organizations, or those of the publisher, the editors and the reviewers. Any product that may be evaluated in this article, or claim that may be made by its manufacturer, is not guaranteed or endorsed by the publisher.

## Supplementary material

Supplementary Video 1 Renal angiography showed focal stenosis in the mid-portion of the right renal artery.

Supplementary Video 2 A bare metal stent was implanted because of dissection after balloon dilation.

Supplementary Video 3 Severe stenosis was found in the right inferior renal branch artery.

Supplementary Video 4 Balloon angioplasty was performed for the branch artery and the lesion vessel was dilated successfully.

Supplementary Video 5 Intravascular ultrasound revealed that eccentric intimal thickening caused stenosis of the branch artery.

Supplementary Video 6 Intravascular ultrasound revealed no neointimal hyperplasia in the stent.
